# Malarial Fever, Accompanied by Diarrhœa and Followed by Inflammation of the Brain

**Published:** 1874-03

**Authors:** J. C. Burroughs

**Affiliations:** Rome, Ga.


### MALARIAL FEVER ACCOMPANIED BY DIARRHŒA AND FOLLOWED BY INFLAMMATION OF THE BRAIN.

BY J. C. BURROUGHS, M.D., ROME, GA.

With no desire on my part to have my name appear in print, save *pro bono publico*, I have written out, at the suggestion of some of my friends, the statement of a case to which, for many weary days and nights, I was an eye witness.

Desiring also, to give honor to whom honor is due, I would state that to my friends, Dr. J. G. Thomas, one of Savannah's most prominent physicians; and to Dr. A. J. Semmes, of whom no other comment is necessary, than to say he it was who immortalized himself in Memphis during the terrible scourge to which that city was subjected the past year—a physician whom Savannah is justly proud of, and Memphis can never do too much reverence to, I am indebted for all that human agencies could have accomplished.

The patient was my own child, nearly one year of age. She was attacked with diarrhœa on the 1st of September, 1873, in Effingham county, near Savannah, where I was at that time in practice, but the attack, not appearing of a serious nature, I left her under the care of her mother who had given her some simple astringent, while I went to see a patient. I returned home about 5 o'clock, P.M., and found the bowels were being constantly acted on, and there being indications for mercury, gave hydrarg, cum creta in one grain doses every hour for six consecutive hours, with no beneficial result, and had her put in a warm bath every six hours. The bowels continued loose all night; there were from forty to fifty evacuations in the twenty-four hours. As there was much mucus, gave

| | |
|---|---|
| ℞.—(*a*). Mucilage, Gum Acaciæ . . . | ℥ iii. |
| Sacch. Albi . . . . . . | ʒ vi. |
| Spir. Ætheris Nitrici . . . . | ʒ iii. |
| Spir. Terebinthinæ . . . . . | ʒ ii. |
| Magnes. Ust. . . . . . . . | gr. xiii. |

M. S. Teaspoonful every 4 hours.

The following morning I sent to Savannah for a physician, and Dr. Thomas responded immediately. As there was fever, he ordered quinine in two grain doses every two hours until the fever was broken, and calomel in one grain doses every hour until four grains were taken. The effect of the quinine was felt after taking eight grains; the irritability of the stomach, after the second dose would permit of but one grain at a time being retained. He also gave for the diarrhœa:

| | |
|---|---|
| ℞.—(*b*). Tinct. Opii Camph. . . . . . | — |
| Tinct. Catechu . . . . . . . | aa ʒ i. |
| Comp. Creta Mixt. . . . . . . | ℥ iv. |

M. S. Teaspoonful every three hours.

(This creta mixture is composed of

| | |
|---|---|
| ℞.—Chalk Mixture . . . . . . . | ℥ v. |
| Aromatic Confection . . . . | ʒ i. |
| Sol. Carb. Ammonia . . . . . . | fl ʒ i. |
| Tinct. Opium . . . . . . . | gtt. xx. |

A tablespoonful of this mixture is a dose.)

This ℞ was taken for two or three days, and at the same time

| | |
|---|---|
| ℞.—(*c*). Camph. Water . . . . . . | ℥ ii. |
| Castor Oil . . . . . . . | ʒ i. |
| Turpentine . . . . . . . | gtt. xv. |
| White Sugar . . . . . . . | ʒ i. |
| Gum Arabic . . . . . . . | ʒ ss. |

M. S. A teaspoonful every two or three hours, as the actions griped her very much with severe straining. At the expiration of this time ℞ *b.* was discontinued and

℞.—(*d*). Subnitrate Bismuth . . . . —
Sacch. Pepsin . . . . . . aa gr. ii.

Substituted. This and ℞. *c.* were given every two hours, alternating. To the latter prescription was added a few drops paregoric, the quantity being reduced upon the head becoming affected. The bowels continuing unchecked, though the number of actions were reduced to about twenty to twenty-five daily, a preparation of krameria and crab's-eyes was now given, of the following proportions:

℞.—(*e*). Oculi Cancor. Pulv. . . . . . ʒ i.

Acaciæ Pulv. . . . . . . . ʒ ii.

Sacch. Albi . . . . . . . ℈ i.

Aquæ Cinnamomi . . . . . . —

Aquæ Puræ . . . . . . aa ℥ iss.

M. S. A teaspoonful every four hours; to which was added:

℞.—(*f*). Tinct. Krameriæ . . . . . gtt. x.

Until the actions become less frequent.

The bismuth and pepsin were kept up, as well as the turpentine preparation, and ℞. *e.* and ℞. *f.*, were given, when the bowels were being acted on too often. This course of treatment continued for four weeks at least; during which the little patient suffered, at times, with follicular stomatitis, which yielded, generally to

℞.—Borate of Soda . . . . . . —
White Sugar . . . . . . aa, qs.

being powdered finely, and with a wet cloth saturated with this, the mouth was well swabbed. At first, this did not appear sufficient, and

℞.—Sodæ Sulphis . . . . . . ʒ i.

Aquæ . . . . . . . . . ℥ iii.

was used in the same manner as the above.

At this time there appeared great cerebral disturbance; the hands were in constant motion, endeavoring, as it seemed, to drive some intangible object from it. The countenance was devoid of that animation and expression which was always visible in health, even in sleep. The bowels being much better than they had previously been, a removal to the city was decided on, and effected without any serious disturbance, under the use of

| | | |
|---|---|---|
| ℞.—Bromide Potassium | . . . . | gr. viii. |
| Syrup Tolu | . . . . . . . | ℨ ii. |
| Water | . . . . . . . | ℨ vi. |

M. S. A tablespoonful every two hours; and blister back of the ears. For two weeks longer this little patient suffered, and at the expiration of that time we saw that the tide had turned; the life which for six weeks had been slowly passing away was now coming back, and we could claim her again as our own.

I have failed to speak of the thermometer, which was daily used, but so frequently changed in its temperature that it would be useless for me to attempt to record it.

Enemas of starch, with four or five drops of laudanum were frequently given during the most severe paroxysms of the disease. The diet consisted chiefly of milk with arrow-root and imperial granum. Beef tea was given with milk, commencing with ten drops and increasing gradually to two teaspoonfuls. Brandy was taken with the food every two hours, in small quantities.

